# Gait Speed is Associated with Executive Function in Latin American Older Adults from LatAmFINGERS

**DOI:** 10.1093/geroni/igaf122.4128

**Published:** 2025-12-31

**Authors:** Myriam Gutiérrez, Alejandra Marroig, Carolina Delgado, Andrea Slachevsky, Ana Charamelo, David Aguillon, Lucía Crivelli

**Affiliations:** Hospital Clínico Universidad de Chile, Santiago, Region Metropolitana, Chile; Universidad de la Republica Uruguay, Montevideo, Montevideo, Uruguay; Hospital Clínico Universidad de Chile, Santiago de Chile, Region Metropolitana, Chile; Hospital del Salvador, Santiago de Chile, Region Metropolitana, Chile; Hospital Británico, Montevideo, Montevideo, Uruguay; Universidad de Antioquia, Medellin, Antioquia, Colombia; FLENI, Buenos Aires, Buenos Aires, Argentina

## Abstract

Dementia prevalence is increasing with aging worldwide, a leading cause of disability in Latin American Countries (LAC). Interestingly, physical performance has been reported as a predictor of cognitive decline, and gait speed has emerged as a potential clinical marker of executive dysfunction. However, evidence of the association between physical and cognitive measures remains scarce, especially in LAC older adults. This study examined the association between gait speed and executive function in older adults from 12 LAC countries. A cross-sectional study including 1,243 participants aged 60 to 77 years from the LatAm-FINGERS randomized multicenter cohort at baseline. Sociodemographic, lifestyle, and health data were collected. Physical performance was assessed using the Short Physical Performance Battery (SPPB), with gait speed measured by a standardized 4-meter walk test. Executive and processing speed were evaluated with the Trail Making Test A and B, Stroop interference index (SII), semantic and phonological fluency, and Concept Shifting Test motor speed (CST-MS). Linear regressions and composite scores were computed. The preliminary results indicate that participants’ mean age was 67.5 ± 4.7 years (67.5% female). Faster gait speed was significantly associated with better Stroop C (p < 0.00001), Stroop P (p = 0.001), semantic fluency (p = 0.02), and higher Mini-Mental State Examination (MMSE) scores. Associations with CST-MS and TMT tests were not statistically significant. This study is the first effort in LAC to associate gait speed with executive tests, contributing to the understanding of a useful functional measure as gait speed, and its potential future preventive applications in LAC.

